# Giant hepatic hemangioma case report: When is it time for surgery?

**DOI:** 10.1016/j.amsu.2020.08.003

**Published:** 2020-08-12

**Authors:** Angelo Amico, Luca Mammino, Stefano Palmucci, Rosalia Latino, Pietro Milone, Giovanni Li Destri, Basile Antonio, Antonio Di Cataldo

**Affiliations:** a General Surgery, Department of Oncological Surgery, University Hospital “Policlinico-Vittorio Emanuele”, 95123, Catania, Italy; b Hospital Umberto I, ASP Enna, 94100, Enna, Italy; c Radiology I Unit, Department of Medical Surgical Sciences and Advanced Technologies “GF Ingrassia”, University Hospital “Policlinico-Vittorio Emanuele”, Catania, 95123, Italy; d General Surgery, Department of Medical Surgical Sciences and Advanced Technologies “GF Ingrassia”, University Hospital “Policlinico-Vittorio Emanuele”, Catania, 95123, Italy

**Keywords:** Liver, Diffusion magnetic resonance imaging, Laparoscopic surgery, Case report

## Abstract

We describe a case of a 30-year-old man who complained intermitted pain in right abdominal flank; a large cavernoumatos hemangioma – up to 6 cm in size – was revealed in the fifth hepatic segment using Ultrasonography and MRI (Magnetic Resonance Imaging).

Indications for treatment – based on imaging features and clinical data – are briefly discussed in our report, providing also a review of existing literature.

## Highlights

• Giant hemangiomas can become an invalidant condition.
• Magnetic Resonance Imaging is the most sensitive radiological technique.
• Giant hemangiomas are suscettibile of a laparoscopic approach

## 1 Introduction

Hemangioma is the most frequent hepatic benignant neoplasia, with autoptic prevalence ranged between 0.4 and 20% and incidence peak between 30 and 50 years [1]. It occurs more frequently in woman – with a female-to-male ratio of 2.5–5:1 – probably due the estrogenic influence. Generally, it is found as a small-sized single lesion, incidentally revealed by Ultrasonography (US), Multidetector Computed Tomography (MDCT) or Magnetic Resonance Imaging (MRI); multiple locations have been reported in a variable range (4–22%) of cases [2,3]. When exceed 4–5 cm in size, some Authors [4,5] have defined lesion as “giant hemangioma”. According to literature, it can reach up to 20–30 cm in main diameter [6]. Very large lesions may be associated with symptoms and complications [7], so that they could require surgical treatments.

However, the surgical management depends not only on the size, but is also conditioned by accurate assessment of location, growth pattern (i.e. exophytic lesion), risk of complications, and anxiety of patients [7,8].

In this article, we describe a case of a young man with giant hepatic hemangioma, incidentally discovered; indications for surgical treatment are briefly discussed, reviewing most relevant articles published in literature.

This article has been reported in line with the SCARE criteria, PROCESS criteria and the following papers:

Agha RA, Borrelli MR, Farwana R, Koshy K, Fowler A, Orgill DP, For the SCARE Group. The SCARE 2018 Statement: Updating Consensus Surgical Case Report (SCARE) Guidelines, International Journal of Surgery 2018; 60:132–136.

Agha RA, Borrelli MR, Farwana R, Koshy K, Fowler A, Orgill DP; SCARE Group. The PROCESS 2018 Statement: Updating Consensus Preferred Reporting Of CAsE Series in Surgery (PROCESS) Guidelines, International Journal of Surgery 2018; 60:279–282.

## 2 Presentation of case

We present a case of a 30 year-old man with a cavernous hemangioma, which was located in the fifth hepatic segment. Patient comes to our attention for oppressive/discontinuous pain located to the right flank and hypochondrium; six months before – he had performed an US examination that revealed a 5 cm liver hyperechoic lobulated lesion.

**Fig. 1:**
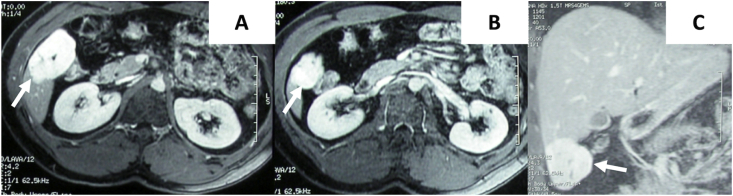
Axial gadolinium-enhanced MR images and Coronal gadolinium-enhanced MR scan of the hemangioma.

Medical history was positive for asthma and nicotine addiction (15 cigarettes/die from 15 years). Physical examination revealed the presence of a tumefaction, painful to deep palpation and not-pulsating, located in right hypochondrium. Laboratory tests were not significant, showing only mild hypercholesterolemia and low level of HDL cholesterol. A MRI examination revealed a normal-sized and regular-shaped liver, confirming the presence of a hemangioma located in the V segment, with measures of 5 × 5 × 6 cm. The hemangioma was highly vascularized, showing globular and centripetal enhancement after gadolinium contrast medium administration: these imaging findings were considered typical features of a cavernomatous hemangioma (Fig. 1). In addition, the lesion showed an exophytic growth, developing from anterior margin of V segment, with a vascular peduncle (Fig. 1).

A surgical treatment was recommended: this therapeutic option was adopted after careful evaluation of several factors, which included lifestyle of patient (he used to do an intense physical activity), lesion measurements and symptoms referred.

**Fig. 2:**
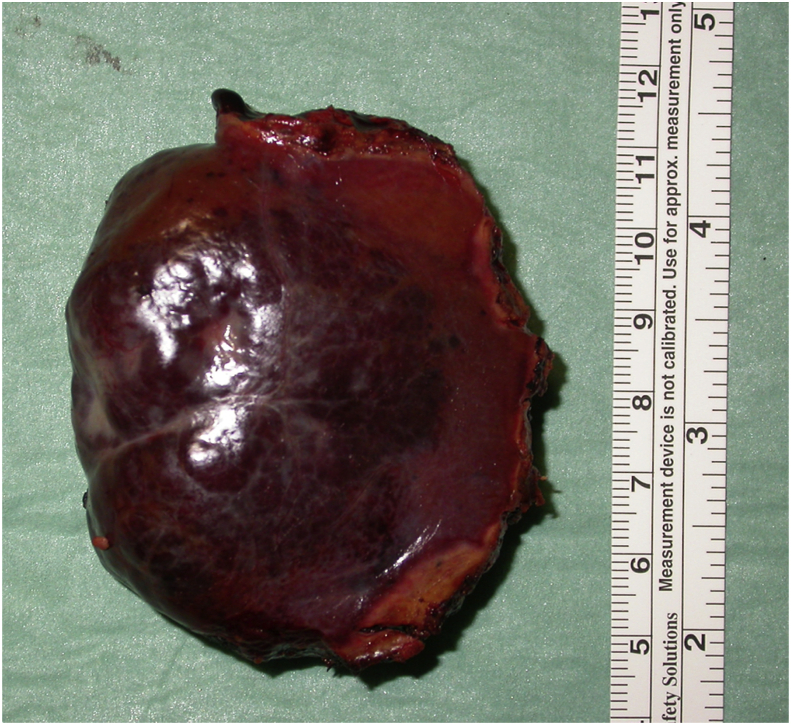
Histological specimen of the hemangioma obtained after excision.

The lesion was totally excised using 4 trocars and an ATLAS 5 mm surgical stapler on the peduncle. The surgical intervention was completed placing a haemostatic gauze and subhepatic surgical drainage. Drainage was removed on second day; no complications were observed. Patient was discharged from hospital on fourth day. Histological examination confirmed hemangioma diagnosis (Fig. 2), with a spongy on-cut appearance, containing multiple white nodular areas. At 5 years from surgery, patient is still in good health.

## 3 Discussion

When hemangioma exceeds 4–5 cm is conventionally defined “giant hemangioma” [1,5]. Microscopically giant hemangioma is composed by a network of vessels coated by single-layer endothelial cells, without capsule but well demarcated from the surrounding hepatic parenchyma. Giant variant has exophytic growth pattern and is generally highly vascularized by arterial branches with slow hematic flow. However, there have been documented atypical forms refilled by severe arterio-venous shunt [9,10].

Several studies have shown lesion size increase in pregnancy or during hormone therapy with estrogen, suggesting a causal role. Glinkova et al. [2] followed in his study 94 women with 181 hepatic hemangiomas, diagnosed with US during 7,3 years [2]. In this study, age at time of menarche was inversely related with hemangiomas sizes (with a p value of 0.0015), whereas age at time of menopause was related with the number of hemangiomas found at first Ultrasonography (p < 0.0001). In the follow-up, an increase of size was shown in 22.7% of women in estrogen therapy versus 9.7% of control. Three variables have been used as prediction of possible growth: ultrasound pattern, number of hemangiomas, hormone therapy. A hypoechoic pattern has been related with size increase, whereas a homogeneous hyperechogenic pattern reduced this risk (p = 0.003). Number of hemangiomas has been inversely related with tumor growth (p = 0.006), and a very strongest correlation was reported for treatment with hormone therapy (p = 0.05).

Unquestionable is the correlation between pregnancy and hepatic hemangioma: according to the experience reported by Fouchard et al. [11], they have documented the growth of hepatic hemangioma in a 29 years old woman – starting from the first pregnancy to the fifth. According to these Authors, estrogen causes vessels ectasia or a specific proliferative trigger [11].

Literature does not provide clear guidelines about “when” and “how” to operate. In symptomless patients or when hemangioma is less than 5 cm, several Authors adopt the “wait and see” strategy, choosing a follow-up over time. Some options may be considered in symptomatic patients or when lesion exceeds 5 cm. Only the 2% of hepatic angiomas are surgical treated, but percentage rises in case of giant angiomas (18–57%) [11]. Many Authors agree on 3 intervention conditions: 1) disabling symptoms because of rapid growth 2) presence of complications 3) doubts about the diagnosis [11]. However these conditions have not unequivocal scientific support.

Hepatic hemangiomas are asymptomatic in 80–86% of cases, however giant hemangiomas are symptomatic in 80–90% of cases. Symptoms are usually, right hypochondrium pain, palpability, right shoulder pain as a sign of fissuring [[5], [6], [7], [8]]. Other symptoms are the result of compression of nearby organs – and namely diaphragm (with respiratory disorders), inferior vena cava, portal vein, hepatic veins (with portal hypertension), biliary tract (with obstructive jaundice), stomach resulting in nausea, vomiting. Rather than chronic symptoms (not always a clear correlation is possible), it has been recommended – as possible indication for surgical treatment – a rapid onset or worsening of preexisting symptoms, which could be considered signs of raising of new complications. The triad of temperature, pain and systemic signs of inflammation is strongly correlated to this occurrence.

In a series of 61 patients, only 7 cases reported evident symptoms – and 6 of this symptomatic subgroup were giant hemangiomas; only 4 patients were surgically treated [12]. Therefore, symptoms may be considered indications for surgery, but if they're not disabling – large size of lesions does not justify preventive surgery, according to the percentage of cases in which is reported spontaneous regression of clinical features.

One of the complications is the Kasaback-Merrit Syndrome characterized by acute thrombosis, entrapment of formed elements and pulmonary embolism, with thrombocytopenia and hypofibrinogenemia [5]. A particular thrombophilia with increase of erythropoietin-like hormone secretion is also related to the hepatic hemangioma. These rare occurrences do not justify surgical treatment, unlike instead of break of lesions. Hemangioma broken represents less than 1% of all hepatic hemangiomas and can be spontaneous or iatrogenic [5,11].

Natural evolution of the hemangiomas is still not well understood, but the possibility of size increment over time influences the surgical choice in about 30% of cases. A large study in a population of 343 patients, followed for an average of 34 months, has shown only 10 hemangiomas increased by 2 cm [[12], [13], [14], [15]]. Ngheim et al. [15] have described 4 cases of giant hemangioma: among these vascular lesions, one increased from 4 cm to 12 cm, and another one from 5 to 9,1 cm in 34 months.

In our case – lesion revealed signs of a space-occupying mass in right hypochondrium, and showed typical radiological features. Therefore, the decision to proceed surgically was based on the following criteria: the presence of worsening symptoms and the risk of dangerous injuries of the lesion. Our decision to perform a laparoscopic treatment was due to the easy anatomic accessibility (V segment, having a wide peduncle). As suggested by several Authors [[16], [17], [18], [19], [20]], laparoscopic surgery is indicated for both lesions with localization in the left hepatic portion and in the lower segments of right lobe. This technique saves more healthy tissue as possible – instead of right or left open hepatectomy. The rare complications intra- and post-operative should put the laparoscopic approach (enucleation, segmental resection) as gold standard in treatment of benignant hepatic lesion, when anatomic accessibility is optimal.

## 4 Conclusion

Indications for treatment of giant hepatic hemangioma – based on imaging features and clinical data – have been briefly discussed in this report. Management and treatment should be based on careful evaluation of clinical and morphological features, and are mainly conditioned by a multidisciplinary approach, in order to act the most safe and non-invasive procedure.

## Ethical approval

Ethical approval:unnecessary.

## Sources of funding

None.

## Author contribution

Angelo Amico: conceptualization;data curation,methodology;writing original draft.

Luca Mammino: data curation;writing-review and editing.

Stefano Palmucci:investigation;supervision;validation.

Rosalia Latino: methodology.

Pietro Milone: validation.

Giovanni Li Destri: methodology.

Basile Antonio: validation.

Antonio Di Cataldo: conceptualization; supervision; validation.

## Research registration number

1. Name of the registry:

2. Unique identifying number or registration ID:

3. Hyperlink to your specific registration (must be publicly accessible and will be checked):

## Guarantor

Angelo Amico

Antonio Di Cataldo

## Consent

Written informed consent was obtained from the patient for publication of this case report and accompanying images. A copy of the written consent is available for review by the Editor-in-Chief of this journal on request.

## Provenance and peer review

Not commissioned, externally peer reviewed.

## Declaration of competing interest

None.
